# A Cooperative Downloading Method for VANET Using Distributed Fountain Code

**DOI:** 10.3390/s16101685

**Published:** 2016-10-12

**Authors:** Jianhang Liu, Wenbin Zhang, Qi Wang, Shibao Li, Haihua Chen, Xuerong Cui, Yi Sun

**Affiliations:** 1 College of Computer and Communication Engineering, China University of Petroleum, Qingdao 266555, China; audiproduct@gmail.com (W.Z.); lishibao@upc.edu.cn (S.L.); chenhaihua@upc.edu.cn (H.C.); cuixuerong@upc.edu.cn (X.C.); 2 Institute of Computing Technology Chinese Academy of Sciences, Beijing 010190, China; wangqi08@ict.ac.cn (Q.W.); sunyi@ict.ac.cn (Y.S.)

**Keywords:** cooperative downloading, digital fountain code, VANET

## Abstract

Cooperative downloading is one of the effective methods to improve the amount of downloaded data in vehicular ad hoc networking (VANET). However, the poor channel quality and short encounter time bring about a high packet loss rate, which decreases transmission efficiency and fails to satisfy the requirement of high quality of service (QoS) for some applications. Digital fountain code (DFC) can be utilized in the field of wireless communication to increase transmission efficiency. For cooperative forwarding, however, processing delay from frequent coding and decoding as well as single feedback mechanism using DFC cannot adapt to the environment of VANET. In this paper, a cooperative downloading method for VANET using concatenated DFC is proposed to solve the problems above. The source vehicle and cooperative vehicles encodes the raw data using hierarchical fountain code before they send to the client directly or indirectly. Although some packets may be lost, the client can recover the raw data, so long as it receives enough encoded packets. The method avoids data retransmission due to packet loss. Furthermore, the concatenated feedback mechanism in the method reduces the transmission delay effectively. Simulation results indicate the benefits of the proposed scheme in terms of increasing amount of downloaded data and data receiving rate.

## 1. Introduction

With the development of wireless communication technology, there is an increasing demand to access the Internet for commuters on vehicles. The goal can be achieved using widely available cellular systems. In some places such as highway scenarios, however, base stations are built with sparse distribution (the two BS are 2.5 km apart in highway scenarios and 400m apart in urban areas), which leads to poor performance. Furthermore, the high cost of a cellular system is another barrier to restrict the development of accessing Internet on vehicles. Wi-Fi is another technology that can be employed by VANET. The disadvantage of Wi-Fi access is the constrained communication area.

Cooperative downloading is an effective way to extend the communication area and improve the amount of downloaded data in VANET. When a client travels out of a hotspot, it can download data from encountering vehicles which carry the data it needs. The method contributes to data sharing and an improvement of the amount of downloaded data. However, due to the poor channel quality and the changing vehicle speeds frequently, the data carried by cooperative vehicles are always lost and cannot be totally transmitted to the client. The problems fail to satisfy the requirement of some applications with high quality of service (QoS). The delay is quite unacceptable when the client reports the loss data to the next hotspot. Although some compensation methods [1] can improve the downloading proportion, frequent data relay occupies the limited bandwidth of VANET.

Digital fountain code (DFC) [2], developed by John Byers, can be applied for the field of wireless transmission to improve channel utilization and transmission efficiency. Fountain code is a kind of rateless code. This means that a potentially limitless number of encoded packets can be generated from the information source. Assuming that the sender encodes the *k* raw data with DFC and generates encoded packets continuously, the receiver will recover the raw data successfully as long as it receives any subcollection of *k*(1 + *ε*) encoded packets. Not only a small decoding overhead *ε* but also DFC has a simple decoding method and low complexity. The biggest difference between DFC and LDPC (Low Density Parity Check Code) is that DFC has no code length. In other words, the code length multiplies towards infinity. However, DFC does not perfectly adapt to DTN (delay tolerant network) because the communication time between the sender and the client is very short, which results in not collecting enough packets to recover the raw data. 

DFC is a technology of error control working on a data link layer. It is appropriate for an erasure channel. In the field of cooperative communication, the source and the destination nodes can match channel capacity self-adaptively by aid of DFC, thus improving transmission efficiency and reliability [3]. However, traditional DFC cannot adapt to the environment of VANET because the nodes move rapidly and the topology changes frequently. This is attributed to two main reasons: (1) Every relay node has to encode and decode data when receiving and sending packets. Obviously, the process greatly increases the burden of the relay nodes and the total computing complexity; (2) When receiving the data, the relay node needs to feedback to the forward node, which decides whether it continues to send the packets or not. The process is inappropriate for VANET because the communicating time among vehicles is so short that the source might leave the communication area of the client when it receives enough data. Furthermore, the mechanism of the cascade feedback brings about a long transmission delay.

**Figure 1:**
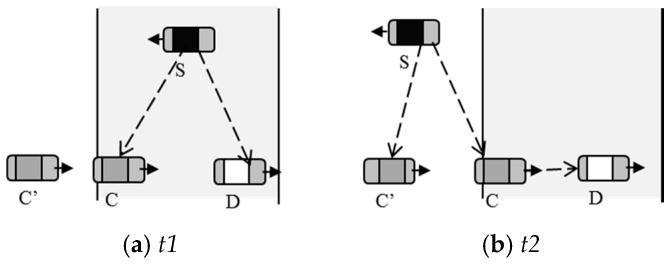
Cooperative downloading using digital fountain code (DFC). (**a**) *S* sends data to *D* directly; (**b**) *C* relays data from *S* to *D* when *S* leaves the communication area of *D*.

To solve the problems above, the paper proposes a cooperative downloading method based on a two-layer distributed DFC. The method makes use of DFC and the mechanism of cooperative downloading to decrease the packet loss rate and to increase communication time, respectively. Figure 1 shows the basic idea of the scheme. The vehicle *S* encodes the raw data into LT-encoded packets before it sends to the client *D* when encountering. During the process of transmitting, another vehicle, *C* or *C*’, running in the communication area of *S*, receives the packets (Figure 1a). The *C* or *C*’ encodes the packets and sends to *D* when *S* leaves the communication area of *D* (Figure 1b). Although some packets are likely to be lost, *D* can restore the raw data once it receives enough packets from *S* and *C*.

The difference between this and other studies is that the cooperative vehicles are not employed to relay original data packets but to transmit DFC encoded packets. The client does not need to receive every packet sent by the source to recover the original data. In other words, packet loss is accepted. The characteristic adapts to VANET with a high packet loss rate. Furthermore, it is possible that the vehicle carrying the raw data leaves the communication area of the client without finishing transmission. As a result, the client cannot send feedback to the senders. Therefore, a distributed fountain code is proposed in the method to solve this problem. The cooperative vehicles do not decode the packets received from the source vehicles, but encode the encoded packets secondly using the distributed function before sending to the client. The method can increase the amount of downloaded data greatly.

## 2. Related Work

Cooperative downloading in vehicular networks was first introduced by Nandan et al. [4] as a part of the protocol-SPAWN for cooperative content retrieval and sharing among users aboard vehicles. MobTorrent [5] improved the amount of downloaded data. The study ignored the packet loss rate and the client had to obtain the loss packets in the next hotspot. Dongyao Jia et al. [6] proposed a retransmission scheme to reduce the packet loss rate at the cost of more retransmission time, which brought about low throughput. Yuchen Wu et al. [7] proposed to exploit trajectory prediction to improve data delivery. However, they did not discuss the solution for the loss of the packets. A compensation method was proposed in [8]. In this method, the loss packets were relayed in cooperative vehicles in order to reduce the packet loss rate. Furthermore, Liu [9] proposed the strategy of data uploading in a VANET-based mobile cloud service for enlarging the communication area and reducing the delay of remote transmission.

DFC is an ideal solution for large-scale data distribution and reliable broadcast. Some studies [10,11] have adopted independent digital fountain code to guarantee the reliability of each hop in the two hops. However, every relay node had to encode and decode data and sent an acknowledge (ACK) to the source node. Obviously, the method brought more computation complicity. Furthermore, frequent information feedback causes a longer delay. In order to solve the problem, cascade coding was proposed in [12]. The relay node did not decode the packets, but encoded the packets secondly after receiving from the source node. The problem was that the cooperative transmission based on the cascade encoding brought about more decoding complicity in the client. Meanwhile, Rui Cao et al. [13] proposed DLT (decomposed LT codes). The two links (from the source to the client and from the relay to the client) in DLT employed the same DFC to keep reliability of communication and reduced computation complicity and delay. However, the model ignored the packet loss rate among the direct link and the other links. It only gave the value span of the distribution function in the first layer, and it was difficult to fulfill. Shabbier Ahmed proposed VANETCODE [14], which divided data into packets before encoding them. Every vehicle passing AP (Access Point) downloads serval packets. They exchange the packets after leaving the communication area of AP. The method has high efficiency when the same data is requested by different users. However, it is not applicable for the requirement that different users hope to obtain different data. Similarly, Hao et al. [15] developed an application layer data sharing protocol that coordinates the vehicles to relay data for sharing according to their positions. Such coordinated sharing can avoid collisions in the medium access control (MAC) layer and the hidden terminal issue in multi-hop transmissions. Distributed-fountain network code (DFNC), which has low encoding, re-encoding, and decoding complexity, is proposed in [16]. However, the decoding process in intermediate vehicles will increase transmission delay.

## 3. The Method of Cooperative Downloading

### 3.1. The Strategy of Cooperative Downloading

To illustrate the method clearly, we assume that only one client requests cooperative downloading and that APs can communicate with each other via the Internet. According to the request of the client, APs choose a group of vehicles to provide cooperative downloading services. Each vehicle was equipped with a Wi-Fi interface and a DSRC protocol stack. Depending on running direction, the vehicles were divided into two groups: one moving with the same direction as the client (represented by $\vec{v}$) and the other moving in the opposite direction (represented by $\overset{\leftarrow }{v}$). When the client left an AP coverage area without finishing its downloading, the next AP chose vehicles in {$\overset{\leftarrow }{v}$} that can catch up with the client and download the remaining packets to it. The principles of choosing cooperative vehicles included no overlapping collision area and a pursuit of maximum throughput. 

**Figure 2:**
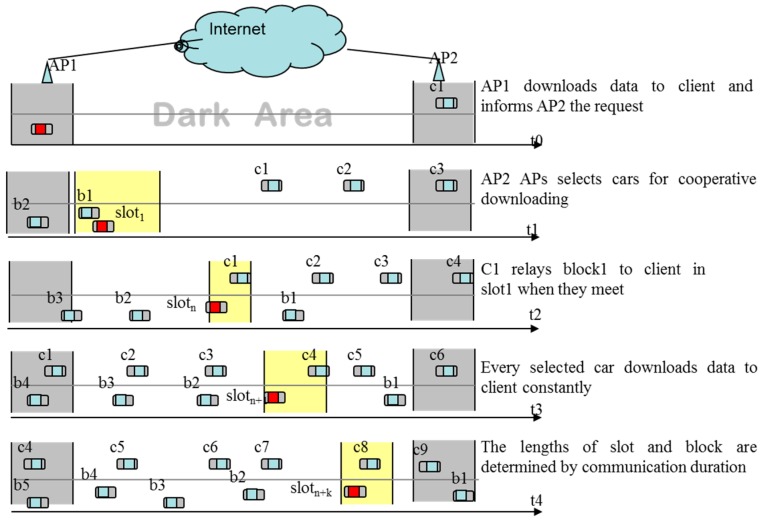
Cooperative downloading based on dynamic slots.

As shown in Figure 2, the vehicles entered and left the AP communication area at different times as well as encountered the client in the dark area (DA) at different times and areas. Therefore, AP could choose a group vehicles to relay data to the client in the DA, as shown at t1–t4 in Figure 2. Due to the limited communication area of the vehicles (about 300 m using DSRC), the amount of data the cooperative vehicles carried was decided by the encountering time with the client. According to previous research [17], traffic conforms to Poisson distribution. When *λ* = 5, up to 20% vehicles can act as the helpers. Transmission collision will take place if more than 20% vehicles provide cooperative downloading services. Therefore, which vehicles are selected to provide cooperative downloading services is closely related to system throughput. According to the characteristic of VANET, we propose a cooperative downloading strategy based on dynamic slots. In this strategy, the DA is divided into several slots according to the encountering time between the vehicles and the client.

While entering an AP coverage area, the vehicle *n* registers its ID, the speed *v_n_*, and the present time *t_n_*. Every AP maintains a list including all vehicles in its coverage area, which is represented by *List* = {(*id*_0_, *v*_0_, *t*_0_), …, (*id_n_*, *v_n_*, *t_n_*)}. The list changes along with vehicles entering or leaving the communication area of the AP. *AP1* and *AP2* exchange their *Lists* every 30 s and the relevant items will be removed when an AP finds that the vehicle with a newer *t* is in the list of other APs. 

When *AP1* receives a downloading request from a client, it searches for the content from the Internet before it downloads the data to the client. The size of the downloading data is determined by its running time in the communication area, which can be calculated according to the information in the *List*. If the downloading is unfinished, *AP1* informs *AP2* of cooperative downloading. 

When *AP2* receives the task, it picks out a group of vehicles that will meet the client in different time slots of the DA and put them in the collection *M* = {(*id*_0_, *v*_0_, *t*_0_, *B*_0_, *E*_0_, *T*_0_), …, (*id_n_*, *v_n_*, *t_n_*, *B_n_*, *E_n_*, *T_n_*)} in items of meeting time. (*id_n_*, *v_n_*, *t_n_*, *B_n_*, *E_n_*, *T_n_*) represent the vehicle ID, its average speed, its register time, the begin time of encountering, the end time of encountering, and the selecting time, respectively. The *slot_n_* = (*E_n_* − *B_n_*) is the entire duration of communication when the client meets the helper *C_n_*. The *B_n_* and *E_n_* can be calculated by Equations (1) and (2).

(1) $$B_{n}=T_{n}+\frac{D+2L-(T_{n}-t_{s})\times v_{s}-(T_{n}-t_{n})\times v_{n}-d}{v_{s}+v_{n}}=\frac{D+2L-d+v_{s}t_{s}+v_{n}t_{n}}{v_{s}+v_{n}}$$

(2) $$E_{n}=B_{n}+\frac{2d}{v_{s}+v_{n}}=\frac{D+2L+d+v_{s}t_{s}+v_{n}t_{n}}{v_{s}+v_{n}}$$

As shown in Equations (1) and (2), *T_n_* has nothing to do with *B_n_* and *E_n_*. If the DA is regarded as a linear space on a time axis, then the *slot_n_* becomes a communication slot in which a cooperative vehicle communicates with the client. The strategy guarantees no overlap of the slots for improving channel utilization. 

Assuming that the bandwidth between vehicles is *W*, the cooperative vehicle *n* should carry the data of *W* × (*E_n_* − *B_n_*) in theory. However, due to the characteristics of bad channel quality and high packet loss in the environment of VANET, cooperative vehicles have to carry fewer data than the theoretical value to compensate packet loss.

The traditional DFC can be utilized to solve packet loss, but VANET cooperative communication consists of serval links and communication time is short between the source and the destination. Therefore, it is difficult to converge the communicating process though feedback from receiver to source. This problem will result in a longer transmission delay. To solve the problem, we propose distributed DFC to relay data among the client and the helpers. Since the relative vehicle speeds running at the same direction is slower and the communicating time is longer, in this method, we only make use of SDCD (same direction cooperative downloading) and employ DFC with a hierarchical feedback mechanism to decrease transmission delay.

### 3.2. The Algorithm of Distributed Digital Foundation Code

**Figure 3:**
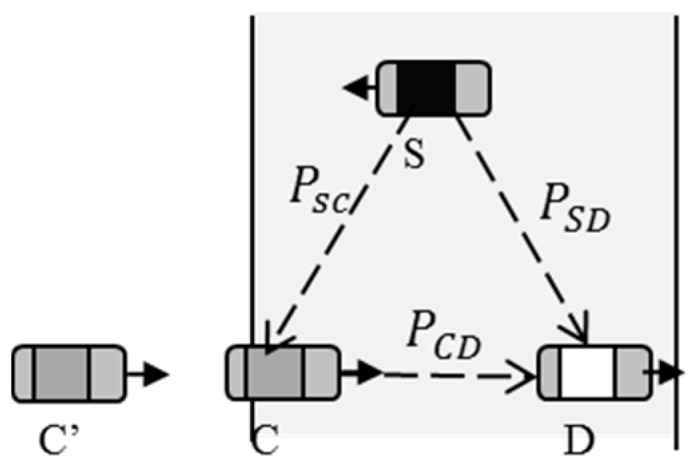
Three-node communication model.

To illustrate the scheme clearer, we turn the two processes in Figure 1 into a three-node communication model. As shown in Figure 3, the original cooperative vehicle *S* encounters the client *D* at its slot in DA and sends encoding packets to *D*. The vehicle *C* running at the same direction with *D* receives these packets and sends data to the client *D* when *S* leaves out the communication area of the client. The source (*S*) and the cooperative vehicle (*C*) stop transmitting packets when they leave out the communication area of the destination (*D*).

Assuming that the channels between nodes are erasure channels, the erasure probabilities are $P_{SC}$, $P_{SC}$, and $P_{CD}$, respectively. *S* and *C* encode the receiving packets into LT-encoded packets. *S* utilizes the degree distributed function $f_{s}\left(x\right)$ to encode the raw data before it broadcasts to *C* and *D*. When *C* receives the encoded packets, instead of decoding, it utilizes another degree distributed function $f_{c}\left(x\right)$ to encode the packets secondly before sending to *D*. *D* employs BP (Belief Propagation) algorithm to decode those encoded packets from S and *C*. If *C* leaves the area of *D*, another cooperative vehicle *C*’ continues to relay the encoded packets. The process does not stop until *D* receives enough packets to restore the raw data. Unlike traditional digital fountain code, *D* does not need to feedback to the original cooperative vehicle because it might have already left the communication area of *S* without receiving enough packets to restore the data. In this method, therefore, *D* only sends feedback to the last senders when finishing transmission. In order to obtain better decoding performance, the key challenge is how to design the degree distributed functions $f_{s}\left(x\right)$ and $\left[f_{c}\left(x\right),\ldots ,f_{c^{n}}\left(x\right)\right]$ so that *D* can easily decode the encoded packets using the degree distributed function θ(x). An algorithm of distributed digital fountain code is given as follows.

Assuming that the source node *S* will transmit *n* packets, it encodes the raw data using DFC with the distributed function $f_{s}\left(x\right)$ before broadcasting the encoded packets to *C* and *D*. The probability of which the packets are received correctly through the link *S*-*D* is (1 − $P_{SD}$), while the probability of which the packets arrive at *C* through the link *S*-*C* is (1 − $P_{SC}$). *C* does not decode the packets, but encodes them secondly using the distributed function $f_{c}\left(x\right)$ before it sends the packets to *D*, and the probability of delivering is (1 − $P_{CD}$). Therefore, via *C*, the probability of which packets are received correctly by *D* is $\left(1-P_{SC}\right)\left(1-P_{CD}\right)$. In this model, there are two ways that the packets are transmitted from *S* to *D*. Assuming that the probability of which *D* receives encoded packets from *C* is *P1*, while the probability of receiving from *S* is *P2*, then

(3) $$P_{1}=\frac{\left(1-P_{SC}\right)\left(1-P_{CD}\right)}{\left(1-P_{SD}\right)+\left(1-P_{SC}\right)\left(1-P_{CD}\right)};$$

(4) $$P_{2}=\frac{\left(1-P_{SD}\right)}{\left(1-P_{SD}\right)+\left(1-P_{SC}\right)\left(1-P_{CD}\right)}.$$

Assuming that the degree distribution function of *S*, *C*, and *D* are $f_{s}\left(x\right)=\sum_{i=1}^{D_{s}}f_{i}x^{i}$, $f_{c}\left(x\right)=\sum_{i=1}^{D_{c}}f_{i}x^{i}$, $\theta \left(x\right)=\sum_{i=1}^{k}\theta_{i}x^{i}$. Obviously, $D_{s}\cdot D_{c}=k$ and all probability of the degree distributed functions are supposed to satisfy $f_{s}\left(x\right)=f_{c}\left(x\right)=\theta \left(x\right)=1,\;f_{s},f_{c},\theta \in \left[0,1\right)$. If the degree distributed function of the packets received by *D* approximately equals the degree distribution function of DFC θ(x), then

(5) $$P_{1}\cdot \left(f_{c}\left(f_{s}\left(x\right)\right)\right)+P_{2}\cdot \left(f_{s}\left(x\right)\right)=\theta \left(x\right).$$

To analyze easily, we employ a matrix formula to represent (5)

(6) δ·ω=θ.

In Equation (6), $\theta ={\left[\theta_{1},\theta_{2},...,\theta_{k}\right]}^{T}$, $\omega ={\left[P_{1}{f_{c}}_{1}+P_{2},P_{1}{f_{c}}_{2}+P_{2},\ldots ,\;P_{1}{f_{c}}_{k}+P_{2}\right]}^{T}$, $\delta ={\left(\delta_{i,j}\right)}_{1\leq i\leq k,1\leq j\leq D_{C}}$, then

(7) $$\delta_{i,j}=\sum \frac{j!}{i_{1}!\ldots i_{D_{fs}}!}{f_{s}}_{1}^{i_{1}}{f_{s}}_{2}^{i_{2}}\ldots {f_{s}}_{k}^{i_{k}}.$$

Because the equation set is nonlinear, it is difficult to obtain an algebraic solution. If we can get a suitable *δ*, *ω* can be determined by linear equation set uniquely. The coefficient of a distributed function of LT coding attenuates with the increase of the degree of distributed function. Therefore, *δ* can be represented by a $D_{f_{c}}\times D_{f_{c}}$ matrix.

According to the fundamental theorem of linear algebra, the necessary and sufficient condition for which *ω* has real solutions is that the rank of *δ* equals the rank of (*δ* *θ*). According to the characteristic of the lower triangular matrix, the rank of δ is $D_{f_{c}}$. Therefore, the rank of (*δ θ*) is also $D_{f_{c}}$, namely,

(8) $$\det\left[\begin{array}{ccc} \delta_{i,1} & \delta_{i,2} & \cdots \\ \vdots & \vdots & \ddots \\ \delta_{i_{D_{fc}},1} & \delta_{i_{D_{fc}},2} & \ldots \end{array}\;\begin{array}{cc} \delta_{i,D_{f_{c}}} & \theta_{i_{1}}\\ \vdots & \vdots \\ \delta_{i_{D_{fc}},D_{f_{c}}} & \theta_{i_{D_{fc}}} \end{array}\right]=0.$$

We deduce the relations further to simplify the formula. Defining $\emptyset_{m}=\left(\delta_{m1}\;\delta_{m2}\;\ldots \delta_{mD_{fc}},\theta_{m}\right)=i_{D_{fc}+1}$. If $\sum_{1\leq m\leq D_{fD}}i_{m}\leq D_{fc}-1$, then

(9) $$\sum_{1\leq m\leq D_{fD}}m\cdot i_{m}\leq D_{fs}\cdot (D_{fc}-1).$$

Combing Equation (5) with (7), we can deduce Equation (10):

(10) $$\delta_{mD_{fc}}\emptyset_{k}-\delta_{kD_{fc}}\emptyset_{m}=\left(0\;\ldots 0\;0\;\delta_{mD_{fc}}\theta_{k}-\delta_{kD_{fc}}\theta_{m}\right).$$

According to Equations (6) and (8), we can deduce Equation (11).

(11) $$\delta_{mD_{fc}}\theta_{k}-{f_{s}}_{D_{fs}}^{D_{fc}}\theta_{m}=0.$$

Assuming that

(12) $$P_{h}=\sum \frac{D_{fc}!}{i_{h+1}!\ldots i_{D_{fs}}!}{f_{s}}_{h+1}^{i_{h+1}}{f_{s}}_{h+2}^{i_{h+2}}\ldots {f_{s}}_{D_{fs}}^{i_{D_{fs}}},$$

$1\leq h\leq (D_{fs}-1)$. According to Equations (7) and (12), we can deduce Equation (13).

(13) $$\delta_{D_{fs}\cdot \left(D_{fc}-1\right)+h,D_{fc}}=D_{fc}{f_{s}}_{D_{fs}}^{D_{fc}-1}{f_{s}}_{h}+P_{h}\;\to \;\frac{{f_{s}}_{D_{fs}}^{D_{fc}}\cdot \theta_{D_{fs}\left(D_{fc}-1\right)+h}}{\theta_{k}}=D_{fc}{f_{s}}_{D_{fs}}^{D_{fc}-1}{f_{s}}_{h}+P_{h}\;\to \;{f_{s}}_{h}=\frac{{f_{s}}_{D_{fs}}^{D_{fc}}\cdot \theta_{D_{fs}\left(D_{fc}-1\right)+h}}{\theta_{k}}-\frac{P_{h}}{D_{fc}\cdot {f_{s}}_{D_{fs}}^{D_{fc}-1}}.$$

Due to $\sum_{h=1}^{D_{fs}}fs_{h}=1$, Equation (14) is deduced from (13).

(14) $$fs_{D_{fs}}=\frac{1}{1+\sum_{h=1}^{D_{fs}-1}\frac{\theta_{k-1}}{D_{fc}-\theta_{k}}}.$$

Therefore, $fs_{h}$ can be obtained by recursion in sequence, while it is easy to solute $fc_{h}$ using the result of $fs_{h}$ and $\theta_{i}$.

In order to compare the delay using DDFC with traditional DFC to download the file in theory, we used the two methods to download the same file. The file was divided into *n* packets and the length of every packet is *l*. The overhead of the decoding of the client was *ε* and source node and the relay node uses degree distributed function $f_{s}\left(x\right)\;\mathrm{and}\;f_{c}\left(x\right)$ to encode the packets, respectively (there is one relay node in this example). The relay node encodes packets directly before sending to the client. Because the relay node does not need to decode data, we can get the transmission time using DDFC as Equation (15):

(15) $$t=\frac{ak\left(1+\varepsilon \right)\left(\frac{W_{n}}{l}\right)+t_{ack}}{k\left(1-P_{SD}\right)\left(1-P_{CD}\right)}.$$

The a is defined as a repeated index. The source node and relay node might send the same packet to the client, so the value of a is 1≥a≥2; $t_{ack}$ is the ACK time which was returned to the relay node by the client when the data were decoded correctly.

Accordingly, using traditional DFC, the relay node has to decode the data received from the source node before re-encoding the data and sending to the client. Furthermore, it needs cascading feedback to the source. These processes increase transmission delay. The delay is as follows:

(16) $$t=\frac{ak\left(1+\varepsilon \right)\left(\frac{W_{n}}{l}\right)\left(1+P_{SD}-P_{SC}-P_{CD}\right)+2t_{ack}}{k\left(1-P_{SD}\right)\left(1-P_{CD}\right)}.$$

Obviously, as shown at Equation (16), although using traditional DFC can reduce the influence of retransmission due to packet loss, frequent encoding and decoding increase processing delay. Therefore, traditional DFC does not adapt to VANET, since the nodes move fast and topology change frequently. Compared with that, DDFC using distributed encoding method reduces the procedure of the encoding and decoding of relay nodes so that the transmission efficiency of cooperative downloading of VANET is greatly improved.

## 4. Performance Evaluation

### 4.1. Simulation Methodology

In this section, we employ a simulation experiment to evaluate the performance of the proposed method. The simulator VanetMobiSim [18] based on JAVA is utilized to conduct the experiments. Our objectives in conducting this evaluation include a data receiving rate of different traffic arrivals, vehicle speed and the changing speed rate, the amount of downloaded data, and the delay of downloading different size files. 

Compared with traditional methods, the DDFC can improve the data receiving rate (DRR) greatly to increase the amount of downloaded data and reduce the delay. Therefore, we regard the DRR as an import index to evaluate the performance of DDFC. The DRR is formally defined as following:

(17) $$\mathrm{DRR}\;=\frac{\sum data\;packets\;received\;by\;the\;destination}{\sum packets\;generated\;by\;the\;source}.$$

**Figure 4:**
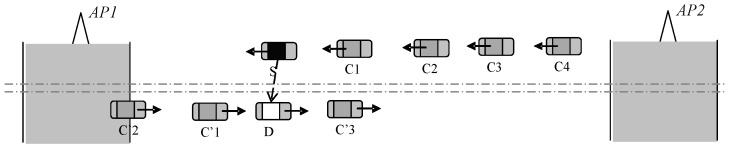
The experiment scenario.

The experiment scenario is shown in Figure 4. The coverage area of AP is set to 800 m, while the communication radius of vehicles is set to 300 m according to [19]. We roughly assume 1 s as overhead to set up connection, 150 Kbytes/s, 200 Kbytes/s, and 50 Kbytes/s as the downloading speed of the APs, and SDCD (same direction cooperative downloading) and ODCD (opposite direction cooperative downloading) driving vehicles, respectively [20]. The range of vehicle speed is set from 60 km/h to 120 km/h. *p* is defined as the changing speed rate in accordance with log-normal distribution [17].

### 4.2. Simulation Results

Firstly, we compared packet loss rates (PLRs) using four different methods (DDFC, DFC-LT, DFC-Relay, and DSRelay). DSRelay [1] does not employ digital fountain code. DFC-LT [13] is the method of transmitting packets from the source to the destination directly using DFC. DFC-Relay transmits packets by aid of cooperative vehicles using traditional DFC. DDFC is the approach proposed in this paper.

**Figure 5:**
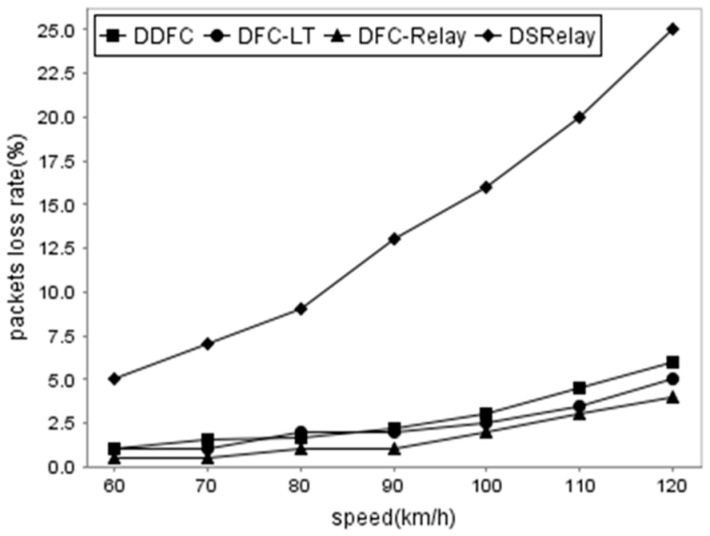
Comparison of packet loss rates using the DDFC, DFC-LT, DFC-Relay, and DSRelay methods.

Figure 5 illustrates the result of when the speeds of vehicles are from 60 km/h to 120 km/h and *p* = 20%, assuming that the traffic arrival follows a Poisson distribution with a rate of one vehicle per *λ* seconds (*λ* = 10). Obviously, PLRs of the four methods rise with the increase in the vehicle speed. DSRelay does not employ DFC. Therefore, the PLR is higher compared with the other methods. Interestingly, when the vehicle speed is 120 km/h, the PLR arrives at 25%. In contrast, the other methods using DFC have a lower rate. Their PLRs keep below 5% when the speed changes from 60 to 120 km/h.

**Figure 6:**
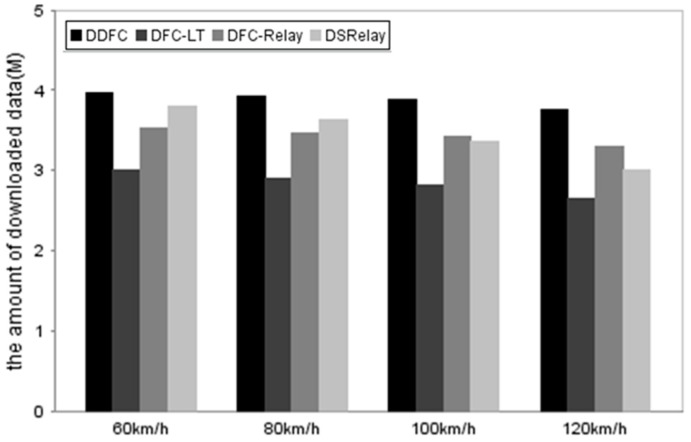
Comparison of the amount of downloaded data using the DDFC, DFC-LT, DFC-Relay, ODCD, and DSRelay methods.

Although there are no obvious differences in PLR using the three methods with DFC, DDFC has better performance in throughput. Figure 6 shows the throughputs using the four methods to download a 4-Mbytes file. The client can download nearly all packets using DDFC. Due to requiring feedback, the amount of data downloaded by the client using DFC-LT and DFC-Relay are about 20% lower than DDFC. In contrast, the amount of data using DSRelay is higher than DFC-LT and DFC-Relay when the vehicle speed is lower than 100 km/h because of cooperative downloading, while the high PLR brings about poor performance when the speed of the client not using DFC is higher than 100 km/h.

The results indicate that, although the PLR of DDFC is a little higher than those of DFC-LT and DFC-Relay, the client can download more data using DDFC compared with the two methods.

Furthermore, we used the four methods to download three files—an 8.1-MBytes file, a 55.3-MBytes video, and a 379.8-MBytes film files. Table 1 shows that the time lengths of downloading the three files are 231 s, 1284 s, and 7980 s using DSRelay. By comparison, DFC-LT and DFC-Relay using the technology of DFC have no obvious advantage to downloading small size files, while the time length drops dramatically when downloading big size files. This is because DFC improves the transmission efficiency and reduces the delay from packet loss. DDFC using cascading feedback displays better performance. The result shows that it only spends 6570 s downloading 379- MBytes files. The transmission efficiency rises by about 20%.

**Figure 7:**
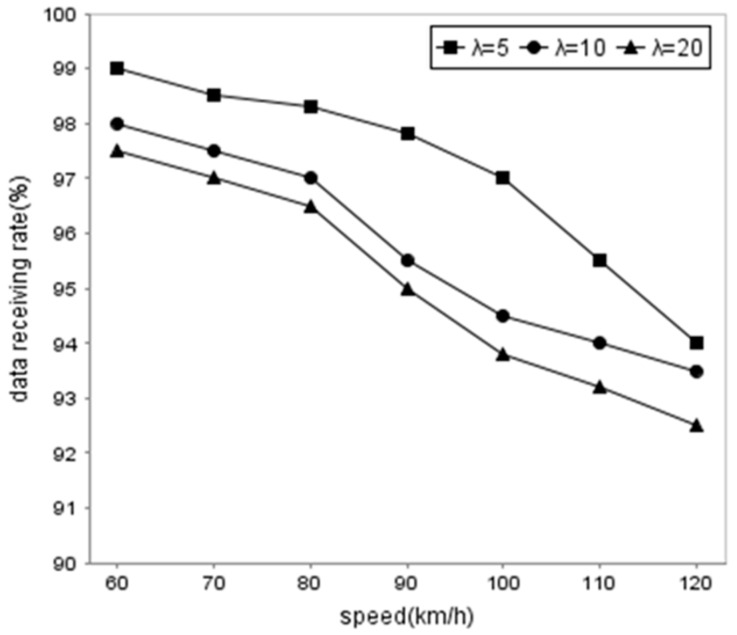
Comparison of data receiving rate generate in different traffic flow, average speed.

**Figure 8:**
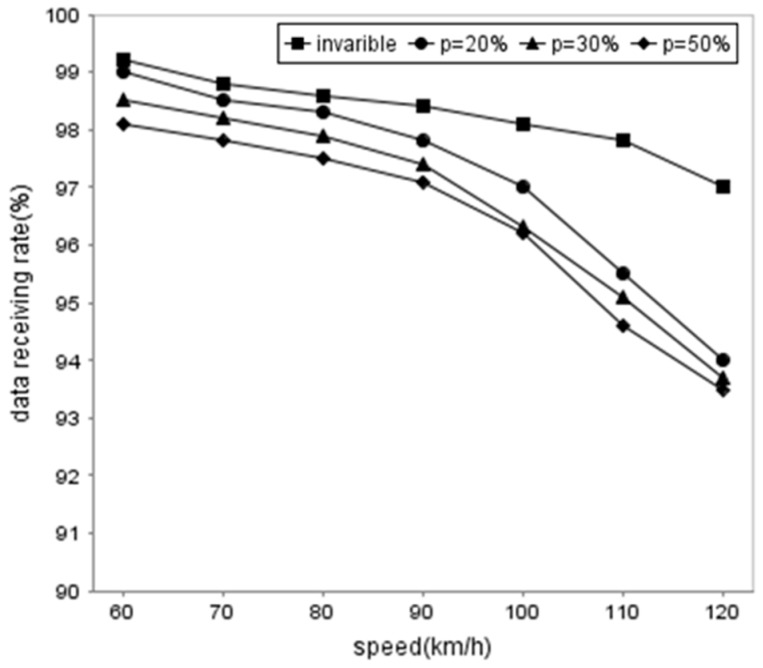
Comparison of data receiving rate generate in different *p* and average speed.

**Figure 9:**
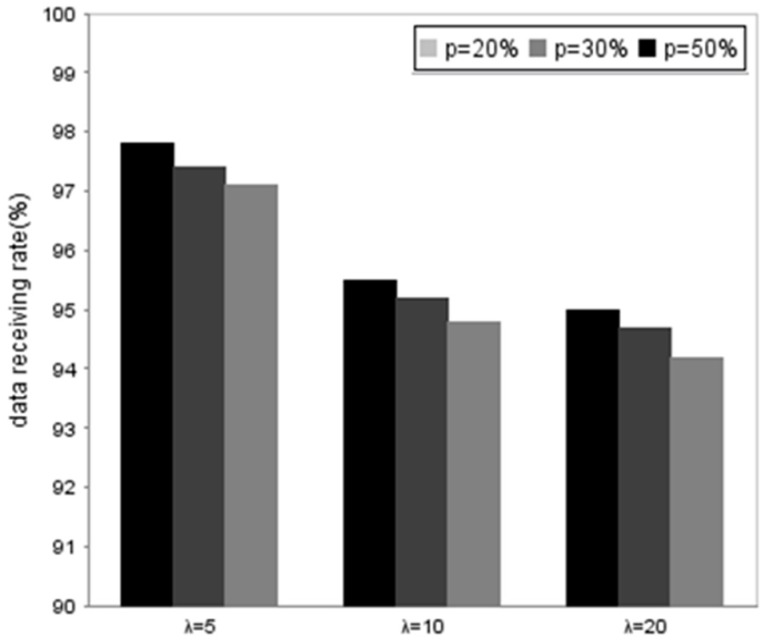
Comparison of data receiving rate generate in different traffic flow and *p*.

In order to validate the performance of the proposed method, we compare the data receiving rate of the vehicles using DDFC in different traffic flow, the changing speed rate, and the different vehicle speeds, respectively. Figure 7 shows that the traffic flow has an influence on DRR because more cooperative vehicles means more opportunities for the client to obtain packets. The DRR is lower when the vehicle speed is faster. Figure 8 and Figure 9 illustrate the influence from the changing speed rate. Obviously, the DRR is higher than 97% when the vehicle speed remains invariable, while the rate drops slightly when *p* = 20%~50% and the vehicle speed is slower than 90 km/h. The DRR decreases rapidly to about 6.5% when the vehicle speed arrives at 120 km/h.

The results indicate that the speed is still an important factor to DRR because of the Doppler effect. Furthermore, traffic flow also influences DRR because more cooperative vehicles result in more communication collision. Besides, the possibility of speed change and DRR are in the inverse ratio according to the results.

## 5. Conclusions

In this paper, we propose the cooperative downloading method for VANET using DFC to increase the amount of downloaded data and enable the transmission to be more robust in a vehicular environment. The requested data is not only directly transferred between an access point and a passing vehicle, but further vehicles running in DA are being used as relays. By making use of DFC, the client can recover the raw data once it receives enough encoded packets from the source vehicle or cooperative vehicles. Besides, the cascading encoding mechanism avoids encoding and decoding processes in relay nodes, which reduces the compute complexity. Simulation results indicate that DDFC can reduce packet loss rate efficiently, while the amount of downloaded data increases by about 20% compared with other DFC methods. It implies that the approach is more robust to occasional packet losses, especially in a highway scenario with low coverage and a high communication cost of cellular systems. In future work, we will continue to research the influence of multi-clients cooperative downloading in multi-channels using distributed DFC.

## Figures and Tables

**Table 1:** Comparison of delay downloading files using the four methods.

Length	DSRelay	DFC-LT	DFC-Relay	DDFC
8.1 MB	231 s	243 s	228 s	225 s
55.3 MB	1248 s	1225 s	1185 s	1108 s
379 MB	7980 s	7690 s	7108 s	6570 s
